# Winter Is Coming: Seasonal Variation in Resting Metabolic Rate of the European Badger (*Meles meles*)

**DOI:** 10.1371/journal.pone.0135920

**Published:** 2015-09-09

**Authors:** David W. McClune, Berit Kostka, Richard J. Delahay, W. Ian Montgomery, Nikki J. Marks, David M. Scantlebury

**Affiliations:** 1 School of Biological Sciences, Institute for Global Food Security, Queen’s University Belfast, Belfast, United Kingdom; 2 National Wildlife Management Centre, Animal and Plant Health Agency, Woodchester Park, Gloucestershire GL10 3UJ, United Kingdom; University of Sassari, ITALY

## Abstract

Resting metabolic rate (RMR) is a measure of the minimum energy requirements of an animal at rest, and can give an indication of the costs of somatic maintenance. We measured RMR of free-ranging European badgers (*Meles meles*) to determine whether differences were related to sex, age and season. Badgers were captured in live-traps and placed individually within a metabolic chamber maintained at 20 ± 1°C. Resting metabolic rate was determined using an open-circuit respirometry system. Season was significantly correlated with RMR, but no effects of age or sex were detected. Summer RMR values were significantly higher than winter values (mass-adjusted mean ± standard error: 2366 ± 70 kJ⋅d^−1^; 1845 ± 109 kJ⋅d^−1^, respectively), with the percentage difference being 24.7%. While under the influence of anaesthesia, RMR was estimated to be 25.5% lower than the combined average value before administration, and after recovery from anaesthesia. Resting metabolic rate during the autumn and winter was not significantly different to allometric predictions of basal metabolic rate for mustelid species weighing 1 kg or greater, but badgers measured in the summer had values that were higher than predicted. Results suggest that a seasonal reduction in RMR coincides with apparent reductions in physical activity and body temperature as part of the overwintering strategy (‘winter lethargy’) in badgers. This study contributes to an expanding dataset on the ecophysiology of medium-sized carnivores, and emphasises the importance of considering season when making predictions of metabolic rate.

## Introduction

Measurements of energy expenditure can provide insights into the form, function and macroecology of organisms [1–3]. Energy expenditure is dependent on a variety of extrinsic and intrinsic factors [4]. In mammals, fundamental measurements of energy metabolism are basal or resting metabolic rate (BMR or RMR). These parameters describe the minimum energy expenditure of an animal at rest and are a function of the cost of somatic maintenance, a key component of the allocation of energy in an organism’s life history [5]. In terms of intrinsic factors, a large proportion of the variation in RMR can be explained by body mass [6], but other important factors include age and sex. Basal metabolic rate or RMR are often higher during periods of elevated growth as experienced by juvenile animals [7], and slow with age [8]. Reproduction in mammals entails a range of direct (e.g. gestation and lactation) and indirect (e.g. remodelling of organ morphology) physiological costs, which consequently pose a range of trade-offs [9]. Hence, different levels of investment by either sex may be reflected as variation in RMR. In terms of extrinsic factors, spatial and temporal differences in RMR are associated with variation in environmental temperature. In temperate climates, most mammals maintain a core body temperature that is warmer than their surroundings and heat loss to the environment must be balanced with heat production [10]. Consequently, seasonal variation in ambient temperature can generate corresponding changes in heat production and also adaptations that manifest as differences in RMR [11, 12].

In the present study we measure RMR of free-living European badgers (*Meles meles*) of differing body mass, age, and sex, across different seasons. Measuring the energy requirements of badgers is relevant ecologically, as they are one of the largest free-living carnivores in the UK and hence affect a number of other species [13, 14]. There have also been relatively few studies on the metabolic rate of wild carnivores and the sample sizes for larger species tend to be small, possibly owing to logistical constraints [15, 16]. We investigate whether age affects RMR by testing if badger cubs had a higher RMR than adults owing to high growth rates during the first year of life [17]. We also examine whether there are sex-related differences in RMR, potentially originating from reproductive investment. In addition, we measure the effects of season, postulating that RMR would be lowest during the winter, as badgers are known to exhibit reduced physical activity [18] and body temperature [19–21] during ‘winter lethargy’ when food intake is reduced [17]. Anaesthesia is often necessary when handling wild animals; however, it is known to affect a wide range of physiological functions including oxygen consumption [22, 23]. Therefore, we also consider its effect on RMR.

Finally, we draw comparisons with allometric predictions for similarly sized mammals to consider badger RMR values in relation to other species. Small mustelid species are thought to have higher metabolic rates for any given body mass (often more than 100% greater) compared with other mammals due to the energetic costs of maintaining a relatively long, thin body. Elevated metabolic rates (approximately 20%) have also been found in large mustelid species [24, 25]. However, BMR has only previously been measured in a single badger and this yielded a relatively low value for a mammal, and a mustelid in particular [25, 26]. This low value could be a consequence of their semi-fossorial lifestyle, which has been linked to low metabolic rates in other mammals [27], or indeed may simply reflect morphological differences. While season is known to influence metabolic rate in a range of species [28, 29], it is rarely controlled for when making allometric predictions of BMR or RMR [30]. Here we examine the phenotypic plasticity of badger RMR in relation to various life-history parameters and season.

## Materials and Methods

### Ethics statement

Permission was granted from Natural England and the UK Home Office to conduct the field research. The protocol was approved by the Food and Environment Research Agency Ethical Review Process prior to commencement.

### Study sites and animals

The study was conducted primarily at Woodchester Park, Gloucestershire, South-West England, where a free-living badger population has been the subject of long-term research. This was supplemented with badgers captured from nearby short-term study areas at Cirencester (approximately 25 km to the east) and Bath (approximately 40 km to the south). During routine capture-mark-recapture studies (see [31]) data were collected from a total of 28 badger social groups. Badgers were caught in steel mesh live-traps which had been pre-baited with peanuts for 4–8 days prior to setting [32]. Captured badgers were then transferred to holding cages for transport to an examination facility with quiet, darkened surroundings in order to minimise stress. Each animal was anaesthetised with two parts butorphanol tartrate (Torbugesic, Wyeth, Ontario, Canada), two parts ketamine hydrochloride (Ketaset, Wyeth, Ontario, Canada) and one part medetomidine (Domitor, Orion Corporation, Espoo, Finland) administered intramuscularly [33]. Individuals were marked with a unique tattoo at first capture, and information on location, sex, age class, and body mass were recorded [32]. Badgers were assigned to one of two age classes: ‘cub’ (in the first year of life up to January the following year); and ‘adult’ (>one year old). Individuals that were trapped between mid-June and mid-September were classed as ‘summer’ samples; those captured between the second half of September and mid-December were classed as ‘autumn’ samples; those caught between the second half of December and the end of January were classed as ‘winter’ samples. Trapping was suspended from February to April inclusive owing to the presence of dependent cubs. As the numbers of captured badgers varied with each trapping event, individuals were initially randomly selected for measurement, although during the later stages of the study underrepresented groups were then targeted. Following examination, recovery from anaesthesia, and RMR measurement, all animals were released at their point of capture. Data were also collected from a captive adult female badger resident at a wildlife rehabilitation centre in Somerset, UK. The study was conducted between July 2009 and January 2013 incorporating data from a total of 57 badgers. The same individuals were not frequently re-captured hence repeated measurements were excluded.

### Resting metabolic rate (RMR)

An open-circuit respirometry system [34, 35] was used to measure RMR. A metabolic chamber (internal dimensions: 49 cm (H) × 47 cm (W) × 102 cm (L)) made of clear Perspex with an internal volume of 235 L was maintained at 20 ± 1°C by a temperature controller (PELT-5, Sable Systems International, Las Vegas, NV, USA). Fresh air from outside was pumped into the chamber by a mass flow meter (FlowKit 100M, Sable Systems International, Las Vegas, NV, USA) at a rate of 46.12 ± 8.57 L⋅min^−1^ (mean ± SD, range: 35–70 L⋅min^−1^) corrected to standard temperature and pressure. The flow rate was adjusted according to the mass of the animal within the chamber in an attempt to maintain oxygen depletion between 0.2–0.8% [36]. Within the chamber, a fan (Proline Mini Fan MF10) ensured thorough mixing and rapid equilibration of the gases inside.

**Fig 1:**
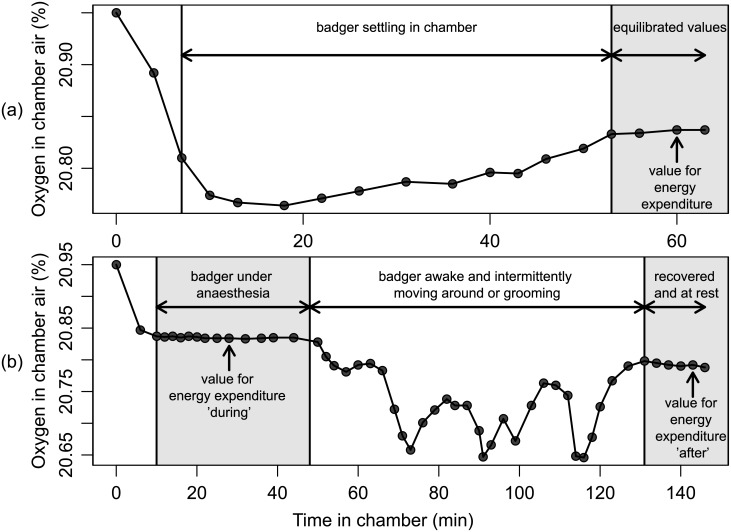
Example traces showing the stabilisation, and effect of anaesthesia on percentage oxygen readings. (a) Oxygen consumption (indicated as % O_2_ in chamber air) over time of an adult male badger after full recovery from anaesthesia (>3 hr). Readings were taken every two to five minutes and show an initial drop of % O_2_ in the chamber and the slow increase in % O_2_ as the badger settles down. The value taken for the calculation of energy expenditure is indicated by the arrow and chosen after sufficient stability had been achieved in the reading for minimum O_2_ consumption. (b) Oxygen consumption over time for an adult female badger placed into the chamber while fully anaesthetised. Readings show the phase during anaesthesia, followed by a waking phase during which the badger intermittently moved or groomed itself, and a final phase of the fully recovered badger while inactive. Readings used to calculate energy expenditure during anaesthesia and after recovery are indicated by arrows and chosen after sufficient stability had been achieved in the minimum O_2_ consumption.

Rate of oxygen consumption ($\dot{\text{V}}{\text{O}}_{2}$) and carbon dioxide production ($\dot{\text{V}}\text{C}{\text{O}}_{2}$) were determined using an oxygen and carbon dioxide analyser (FoxBox Field Gas Analysis System, Sable Systems International, Las Vegas, NV, USA) which sub-sampled dried air (Drierite Laboratory Gas Drying Unit, W.A. Hammond Drierite Co. Ltd., OH, USA) from the metabolism chamber at a rate of 500 ml⋅min^−1^. The analyser was calibrated to 20.95% O_2_ (outside air) prior to the measurement of each animal. Percent O_2_ and CO_2_ were recorded every five minutes after a badger was placed inside the chamber within a holding cage, allowing it to become accustomed to the experimental conditions (most animals lay down immediately). Subsequently, readings were taken every one to three minutes until a stable value was obtained (Fig 1a, time until stable reading: 86.86 ± 44.35 min (mean ± SD)). Badgers were then removed from the chamber, which was flushed with fresh air at a flow rate of 85 L⋅min^−1^ for 15 min which was sufficient time to allow the levels of O_2_ and CO_2_ to return to atmospheric readings. The chamber was cleaned with a disinfectant before the next animal was measured. Both $\dot{\text{V}}{\text{O}}_{2}\;(\text{ml}\cdot {\text{min}}^{-1})$ and $\dot{\text{V}}\text{C}{\text{O}}_{2}\;(\text{ml}\cdot {\text{min}}^{-1})$ were calculated accounting for dilution and concentration effects:

$$\dot{V}O_{2}=\frac{FR\cdot (\left(F_{i}O_{2}-F_{e}O_{2}\right)-F_{e}O_{2}\cdot \left(F_{e}CO_{2}-F_{i}CO_{2}\right))}{\left(1-F_{e}O_{2}\right)}$$ (1)

$$\dot{V}CO_{2}=\frac{FR\cdot (\left(F_{e}CO_{2}-F_{i}CO_{2}\right)-F_{e}CO_{2}\cdot \left(F_{i}O_{2}-F_{e}O_{2}\right))}{\left(1-F_{e}CO_{2}\right)}$$ (2)

where FR is incurrent flow rate (ml⋅min^−1^), F_e_O_2_ is excurrent O_2_ fraction, F_i_O_2_ is incurrent O_2_ fraction, F_e_CO_2_ is excurrent CO_2_ fraction and F_i_CO_2_ is incurrent CO_2_ fraction [37]. Any drift in the analyser was assumed to be linear for baseline correction. The respiratory quotient (RQ) was calculated as the ratio of $\dot{\text{V}}\text{C}{\text{O}}_{2}$ to $\dot{\text{V}}{\text{O}}_{2}$. Energetic equivalents were calculated by multiplying $\dot{\text{V}}{\text{O}}_{2}$ values by the oxyjoule conversion factor of [15.97 + 5.164⋅RQ] J⋅ml^−1^ [38].

### Effect of anaesthesia on RMR

To assess the effect of anaesthesia on RMR, measurements were taken during three distinct phases: (1) ‘before’ anaesthesia ($\dot{\text{V}}{\text{O}}_{2}$ prior to injection of anaesthetic agents); (2) ‘during’ anaesthesia (whilst the animal was recumbent); and (3) ‘after’ apparent recovery from anaesthesia. The latter measurement was taken after at least two to three hours after the badger had initially woken up and was able to move/stand (Fig 1b). For each animal, these three measurements were taken on the same day.

### Effect of ambient temperature on RMR

We measured the metabolic rate of a captive badger at a range of ambient temperatures: 15, 19, 20, and 23°C. Measurements were carried out in January between approximately 12:00 and 17:00, during the period of minimal activity [39]. The badger was placed in the respirometry chamber where it was allowed to become accustomed to its surroundings. The flow rate was 40 L⋅min^−1^. Beginning at the lowest temperature (15°C), percent O_2_ and CO_2_ were recorded every one to three minutes until a stable value was obtained (as above, time until stable reading: 39.75 ± 18.75 min (mean ± SD)). The temperature of the chamber was then increased to the next increment (i.e. from 15 to 19°C) and the animal was then allowed to settle. We then took measurements every one to three minutes until a stable value was obtained. These procedures were repeated for each of the above temperatures to estimate the range of the thermoneutral zone (TNZ).

### Comparison of RMR with allometric predictions

Resting metabolic rate measurements were compared with the allometric prediction for large mustelid species (weighing 1 kg or greater) from Iversen (1972) [25] (Eq 3) and for mammals following Kleiber (1961) [40] (Eq 4):

$$M=84.6\cdot W^{0.78}$$ (3)

$$M=70\cdot W^{0.75}$$ (4)

In Eqs 3 and 4, *M* is basal metabolic rate (BMR) in kcal⋅d^−1^ and *W* is body mass (kg). Measurements were then compared with the prediction from White and Seymour (2003) [41] (Eq 5) which accounts for the variation associated with body temperature, digestive state, and phylogeny. Comparisons were also made with the prediction from McNab (2008) [6] (Eq 6) for carnivores.

$$M=4.17\cdot W^{0.68}$$ (5)

$$M=3\cdot W^{0.752}$$ (6)

In Eqs 5 and 6, *M* is BMR (ml O_2⋅_h^−1^) and *W* is body mass (g). When deriving energetic equivalents from other studies, an oxyjoule conversion factor of 19.9 kJ⋅L O_2_ corresponding to an RQ of 0.76 (the average RQ from the present study) was used if possible.

### Statistical analyses

All analyses were performed using R version 3.2.1 [42]. The lower critical temperature (LCT) was estimated as the temperature at which RMR values ceased to decrease with increasing temperature. The effects of anaesthesia on RMR were examined using a linear mixed effects model (RMR (kJ⋅d^−1^) ∼ body mass + anaesthesia state + badger ID {random}) with the ‘nlme’ package [43], followed by a Tukey contrasts *post hoc* test using the ‘multcomp’ package [44]. A stepwise Akaike’s Information Criterion (AIC) approach was adopted for model selection to investigate the effects of age, sex, and season on body mass. The effect of body mass on RMR (kJ⋅d^−1^) was examined by linear regression. To investigate the effects of age, sex, and season on RMR (kJ⋅d^−1^), multiple analysis of covariance (ANCOVA) models were fitted with body mass as a covariate. Resting metabolic rate values were compared with the various allometric predictions using a Welch corrected one-way analysis of variance (ANOVA), followed by a Dunnett’s many-to-one contrasts *post hoc* test [45]. Cubs were excluded from comparisons with allometric predictions, as juveniles of other mustelid species have previously been found to have elevated metabolic rates due to growth [7]. The percentage difference of RMR between seasons was calculated by dividing the absolute difference between mass-adjusted means by their arithmetic mean. The percentage error of allometric predictions was calculated by dividing the difference between measured and predicted values by the predicted value. Normality and variance assumptions were checked using Shapiro–Wilk and Levene’s tests, respectively. Statistical significance was accepted at *p* ≤ 0.05. Body mass is reported as the mean ± standard deviation. Resting metabolic rate values are reported as the mass-adjusted mean ± standard error (using the ‘effects’ package [46]) of the energetic equivalents unless otherwise stated.

## Results

### Effects of ambient temperature and anaesthesia on RMR

**Fig 2:**
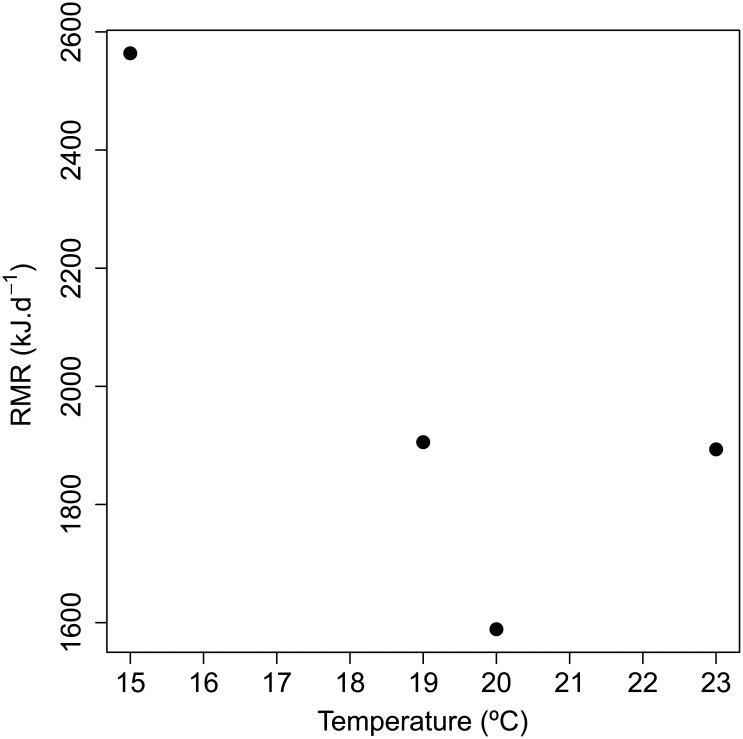
The effect of ambient temperature on RMR in a European badger (*Meles meles*). Metabolic rate of a single adult captive badger in winter, measured during a single session over a range of ambient temperatures. Metabolic rate was highest at 15°C (whole-animal non-adjusted: 2564 kJ⋅d^−1^), and decreased with increasing temperature until 20°C (1589 kJ⋅d^−1^) with a difference of 975 kJ⋅d^−1^. Thus 20°C was deemed to be within the TNZ. Finally, when ambient temperature was raised to 23°C metabolic rate increased slightly to 1893 kJ⋅d^−1^.

**Fig 3:**
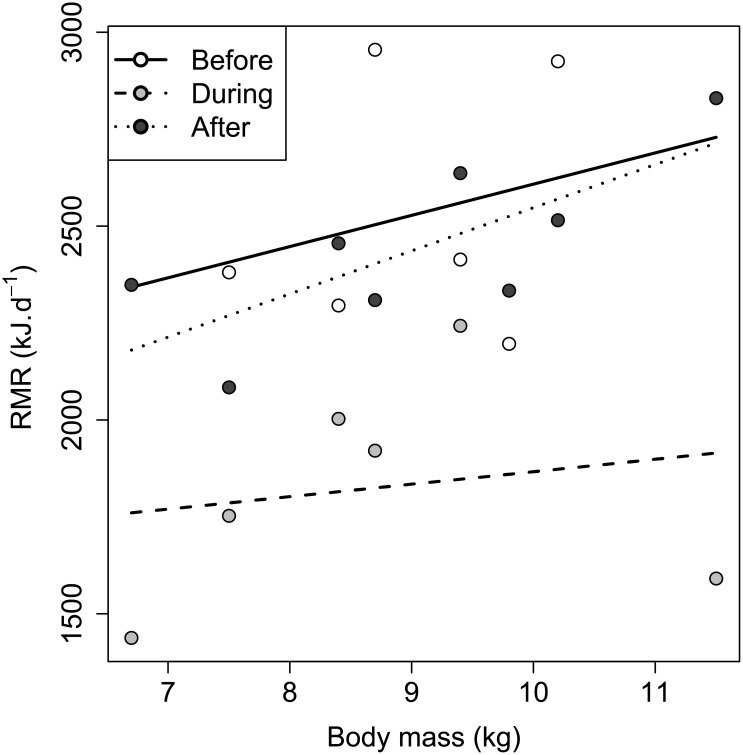
The effect of anaesthesia on RMR in the European badger (*Meles meles*). Comparison of RMR (kJ⋅d^−1^) from 8 adult badgers ‘before’, ‘during’, and ‘after’ anaesthesia (intramuscular administration of two parts butorphanol tartrate, two parts ketamine hydrochloride and one part medetomidine). There was a significant depression in RMR during anaesthesia compared with measurements taken before (*p* < 0.001) and after (*p* < 0.001). However, between two to three hours after waking up (‘after’) there was no longer a significant effect (*p* = 0.8).

Metabolic rate was found to decrease with increasing temperature until 20°C, which was deemed to be within thermoneutrality (Fig 2). There was a significant effect of anaesthesia on RMR (F_2,10_ = 11.9, *p* = 0.0023; before: 2522 ± 108 kJ⋅d^−1^, n = 6; during: 1841 ± 109 kJ⋅d^−1^, n = 6; after: 2431 ± 94 kJ⋅d^−1^, n = 8; adult badgers n = 8). There was no interaction between body mass and anaesthesia state (*p* = 0.74). *Post hoc* tests indicated that there were significant reductions in RMR during anaesthesia compared with both before (*p* < 0.001) and after (*p* < 0.001) anaesthesia. In contrast, RMR measurements taken before and after anaesthesia did not differ from one another (*p* = 0.8; Figs 1 and 3). Thus, for the purposes of analysis measurements recorded before and after anaesthesia were combined.

### Relationship between RMR, body mass, sex, age, and season

There were significant effects of age, sex, and season on body mass in the final model (main effects: F_1,49_ = 20.2, *p* < 0.001; F_1,49_ = 4.8, *p* = 0.034 and F_2,49_ = 6.1, *p* = 0.0043, n = 56, respectively). Importantly, there was also a significant interaction between age and season on body mass (F_2,49_ = 4.2, *p* = 0.021) suggesting that body mass was affected differently by season depending on the age of the animal. Body mass was found to increase with age category (cubs: 5.67 ± 2.97 kg, n = 7, adults: 8.84 ± 1.90 kg, n = 49), and males (9.11 ± 2.06 kg, n = 28) were heavier than females (7.77 ± 2.35 kg, n = 28). *Post hoc* testing (Tukey’s Honest Significant Difference) revealed that badgers were significantly heavier in winter (9.61 ± 1.98 kg, n = 19) in comparison to the summer (7.78 ± 2.57 kg, n = 26; *p* = 0.0071) and autumn (8.01 ± 1.16 kg, n = 11; *p* = 0.023). This was primarily due to differences in the body mass of cubs. Cubs during summer weighed significantly less than adult badgers (3.58 ± 0.38 kg, n = 4; *p* < 0.001), but by autumn (5.70 kg, n = 1; *p* = 0.51) and winter (9.85 ± 0.35 kg, n = 2; *p* = 1) they had reached comparable values. There was no significant difference in adult body mass between seasons (summer-autumn: *p* = 0.51; winter-autumn: *p* = 0.3; winter-summer: *p* = 0.37).

**Fig 4:**
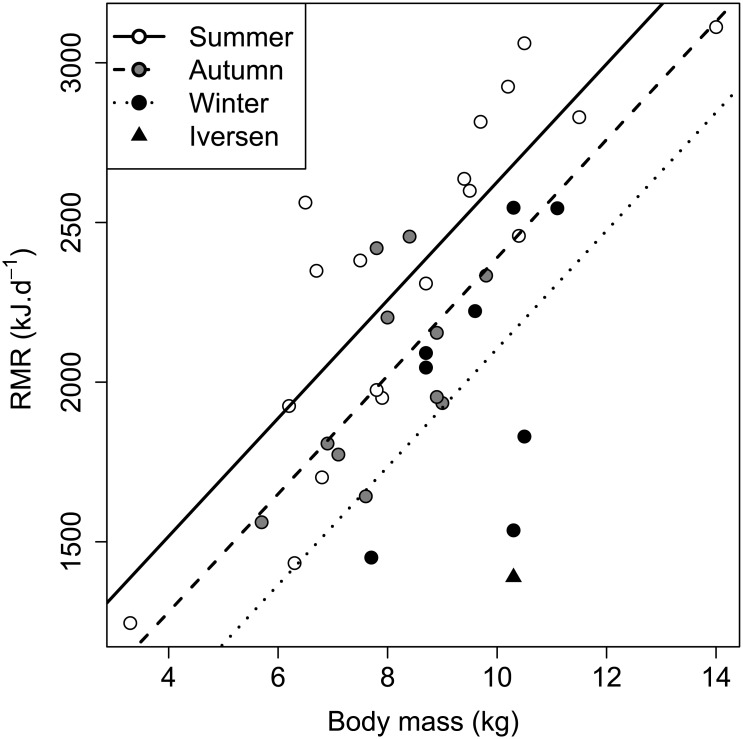
Variation of RMR with season in the European badger (*Meles meles*). Data from 37 wild badgers. The mass-adjusted mean ± standard error summer RMR was 2366 ± 70 kJ⋅d^−1^, n = 18. This was higher than, but not significantly different to the mean autumn RMR (2129 ± 91 kJ⋅d^−1^, n = 11, *p* = 0.11). Resting metabolic rate in winter was significantly lower than in summer (1845 ± 109 kJ⋅d^−1^, n = 8, *p* < 0.001), but not significantly different to autumn (*p* = 0.13). The regression lines are of the values predicted by the ANCOVA model (RMR (kJ⋅d^−1^) ∼ body mass + season) coefficients. The badger BMR value from Iversen (1970, 1972) [25, 26] is also shown for comparison (▴) with a value of 1389 kJ⋅d^−1^.

The most important factor affecting RMR was body mass with larger animals tending to have higher RMR than smaller animals. The least squares regression:

$$\text{RMR}\;\left(\text{kJ}\cdot d^{-1}\right)=766+164.93\cdot \text{body}\;\text{mass}\;\left(\text{kg}\right)$$ (7)

accounted for 45.8% of the total variation (F_1,35_ = 29.6, *p* < 0.001, n = 37). After accounting for body mass, there were no effects of sex or age on RMR (F_1,34_ = 0, *p* = 0.94, and F_1,34_ = 0.2, *p* = 0.63, n = 37, respectively). Conversely, there was a significant effect of season (F_2,33_ = 8.5, *p* = 0.0011, n = 37; Fig 4). There was no interaction between body mass and season on RMR (*p* = 0.98). The mean summer RMR was 2366 ± 70 kJ⋅d^−1^, n = 18. This was not significantly different to RMR in autumn (2129 ± 91 kJ⋅d^−1^, n = 11, percentage difference: 10.5%) after Tukey contrasts *post hoc* testing (*p* = 0.11). Resting metabolic rate in winter was significantly lower than in the summer (1845 ± 109 kJ⋅d^−1^, n = 8, *p* < 0.001, percentage difference: 24.7%), but not significantly lower than RMR in the autumn (*p* = 0.13, percentage difference: 14.3%; Table 1).

**Table 1:** Body mass and RMR for 37 European badgers (*Meles meles*) during summer, autumn, and winter. (Mean ± SD, whole-animal RMR values are non-adjusted.)

	**Summer cubs**	**Summer adults**	**Autumn cubs**	**Autumn adults**	**Winter adults**
Body mass (kg)	3.30	8.80 ± 2.15	5.70	8.24 ± 0.92	9.61 ± 1.15
Whole-animal RMR (kJ⋅d^−1^)	1246	2413 ± 484	1562	2068 ± 286	2033 ± 413
Mass-specific RMR (kJ⋅kg^−1^⋅h^−1^)	15.7	11.6 ± 1.9	11.4	10.5 ± 1.4	8.8 ± 1.5
Whole-animal RMR (ml O_2_⋅h^−1^)	2595	5088 ± 1025	3216	4340 ± 642	4198 ± 839
Mass-specific RMR (ml O_2_⋅kg^−1^⋅h^−1^)	786.4	588.6 ± 98.6	564.2	528.5 ± 70.4	437.7 ± 74.3
Whole-animal RMR (ml CO_2_⋅h^−1^)	2027	3734 ± 770	2654	3261 ± 348	3424 ± 759
Mass-specific RMR (ml CO_2_⋅kg^−1^⋅h^−1^)	614.2	431.0 ± 65.5	465.5	399.0 ± 52.3	356.0 ± 66.2
RQ	0.78	0.74 ± 0.05	0.83	0.76 ± 0.05	0.81 ± 0.07
n	1	17	1	10	8

### Comparison of RMR with allometric predictions

The RMR values measured resulted in the equations:

Summer:

$$M=516.12\cdot W^{0.71}$$ (8)

Autumn:

$$M=546.19\cdot W^{0.63}$$ (9)

Winter:

$$M=300.90\cdot W^{0.84}$$ (10)

All seasons combined:

$$M=586.59\cdot W^{0.61}$$ (11)

**Fig 5:**
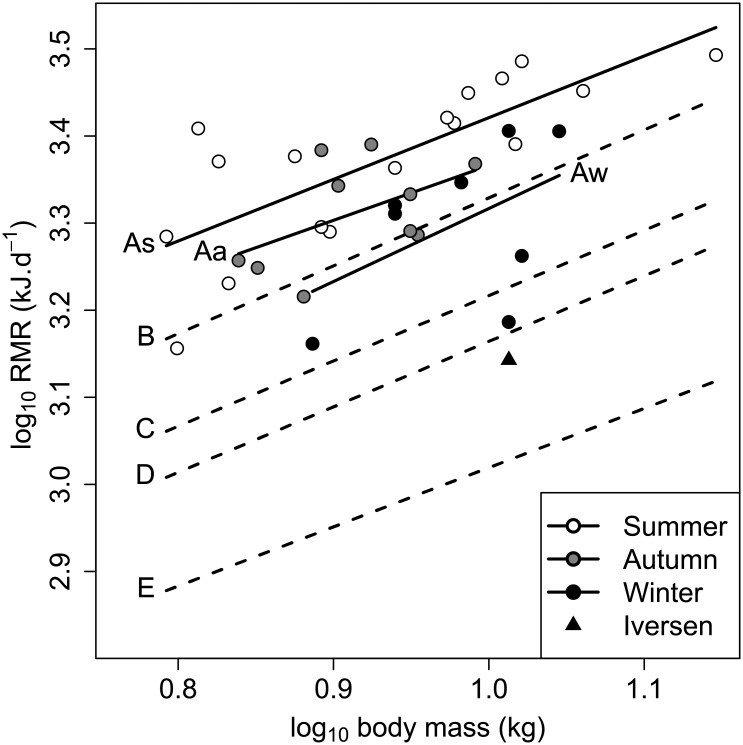
Comparison of RMR with allometric predictions. Regression of log_10_ RMR (kJ⋅d^−1^) against log_10_ body mass (kg) for 35 adult badgers. The solid lines (As, Aa, and Aw) denote the least squares regressions of RMR against body mass in summer, autumn, and winter, respectively. (B) Predicted BMR values from Iversen (1972) [25] (Eq 3) for large mustelid species (weighing 1 kg or greater), (C) Kleiber (1961) [40] (Eq 4) for mammals in general, (D) McNab (2008) [6] (Eq 6) for carnivores, and (E) White and Seymour (2003) [41] (Eq 5) for mammals accounting for body temperature, digestive state and phylogeny are included for comparison. The badger BMR value from Iversen (1970, 1972) [25, 26] is also shown (▴).

In each case, *M* is RMR (kJ⋅d^−1^), 516.12, 546.19, 300.90, and 586.59 are the mass scaling coefficients (base 10 antilog of the intercept), *W* is body mass (kg), and 0.71, 0.63, 0.84, and 0.61 are the mass scaling exponents (slope). There were significant differences between the various predictions of metabolic rate and the measured values (summer: F_4,38.6_ = 55.1, *p* < 0.001, n = 17; autumn: F_4,21.6_ = 103.4, *p* < 0.001, n = 10; winter: F_4,16.8_ = 62.9, *p* < 0.001, n = 8; all seasons combined: F_4,81.8_ = 147.2, *p* < 0.001, n = 35). *Post hoc* testing revealed that the estimated RMR values were higher than predicted by all allometric equations with the exception of the Iversen (1972) [25] (Eq 3) prediction during the autumn and winter (Table 2, Fig 5). Resting metabolic rate values during the summer were significantly different to the prediction of Iversen (1972) [25] (Eq 3) with a mean percentage error (± SD) of 26.1 ± 17.7% (range: −3.7 to 68.1%; *p* = 0.0047). Conversely, RMR values measured in the autumn and winter were not significantly different to the same prediction (autumn: mean percentage error: 13.0 ± 14.0%, range: −4.6 to 37.7%, *p* = 0.076; winter: mean percentage error: −1.7 ± 16.9%, range: −29.6 to 16.7%, *p* = 0.99). When summer, autumn, and winter values were combined, RMR was significantly greater than predicted (mean percentage error: 16.0 ± 19.7%, range: −29.6 to 68.1%, *p* = 0.0037).

**Table 2:** Comparison of European badger (*Meles meles*) RMR (kJ⋅d^−1^) measured during summer, autumn, and winter (n = 35 adults) with allometric predictions.

**Prediction**	**Season**	**Mean error ± SD (%)**	**Error range (%)**	**Significance**
Iversen (1972) [25] (Eq 3)	Summer	26.1 ± 17.7	−3.7–68.1	S (*p* = 0.0047)
	Autumn	13.0 ± 14.0	−4.6–37.7	NS (*p* = 0.076)
	Winter	−1.7 ± 16.9	−29.6–16.7	NS (*p* = 0.99)
	Combined	16.0 ± 19.7	−29.6–68.1	S (*p* = 0.0037)
Kleiber (1961) [40] (Eq 4)	Summer	62.6 ± 22.6	23.0–114.9	S (*p* < 0.001)
	Autumn	45.5 ± 18.0	22.5–77.0	S (*p* < 0.001)
	Winter	27.2 ± 21.9	−8.8–51.2	S (*p* = 0.025)
	Combined	49.6 ± 25.2	−8.8–114.9	S (*p* < 0.001)
White and Seymour (2003) [41] (Eq 5)	Summer	153.4 ± 34.5	87.7–228.6	S (*p* < 0.001)
	Autumn	126.0 ± 27.8	89.4–174.1	S (*p* < 0.001)
	Winter	99.8 ± 34.7	44.1–138.8	S (*p* = 0.0012)
	Combined	133.3 ± 38.7	44.1–228.6	S (*p* < 0.001)
McNab (2008) [6] (Eq 6)	Summer	83.5 ± 25.5	39.0–142.7	S (*p* < 0.001)
	Autumn	64.2 ± 20.3	38.3–99.8	S (*p* < 0.001)
	Winter	43.5 ± 24.7	2.9–70.6	S (*p* < 0.001)
	Combined	68.9 ± 28.4	2.9–142.7	S (*p* < 0.001)

S = significant, NS = not significant.

## Discussion

### Variation in RMR

#### Season

Resting metabolic rate was significantly higher during summer than winter. This concurs with previous studies that indicate a reduction in body temperature in badgers during cold winter months in order to conserve energy and rely on fat reserves [19–21]. The observed change in metabolic rate in this study might be explained by annual variation of the thyroid hormone thyroxine. Thyroxine is known to increase BMR in humans [47, 48], and is associated with hibernation in other species such as the European hedgehog (*Erinaceus europaeus*) and black bear (*Ursus americanus*) [49, 50]. High thyroxine utilisation rates have also been suggested to explain the unusually high BMR of the shrew (*Sorex vagrans*) [51]. In badgers, blood plasma concentrations of thyroxine have previously been shown to fall rapidly to low levels by December until a rise occurs during March [52]. Thus, a winter depression in thyroxine would correlate with the reduction in metabolic rate seen in the current study. In addition, relatively high levels of thyroxine have been shown to coincide with the onset of moulting and maximal hair growth in adult badgers [53]. Moulting is an energetically costly process in other species [54, 55], which may also contribute to the seasonal RMR differences in this study.

It is possible that seasonal differences in metabolic rate vary across the wide geographic distribution of badgers, as does the extent of winter lethargy [18]. Badgers are known to undergo seasonal changes in body mass largely due to the accumulation of subcutaneous fat reserves [56], which may also be of significance given that both fat mass and fat-free mass contribute separately to BMR [57, 58]. Higher summer BMR has been reported in other mustelid species, the American mink (*Neovison vison*), and Siberian polecat (*Mustela eversmannii*) [59, 60].

#### Age and sex

We did not observe age related differences in RMR, which was unexpected as juvenile animals generally experience elevated levels of growth [7, 61], and badger cubs are known to gain body mass and moult continuously [17, 62]. Further work with a larger number of cubs may reveal differences. We also found no difference in RMR between males and females. This may be because we did not trap badgers from February to May (for welfare reasons), which is when most cubs are born and suckled [63] and, therefore, when high energetic costs in females might be expected. Although pregnancy commonly occurs from December to February [17], which was within our measurement period, it has previously been found that gestation in badgers is not associated with a loss in body condition, in contrast to lactation [64]. In the American badger (*Taxidea taxus*), the energy requirements of gestation are thought to be relatively small, and up to 16 times less than those of lactation [65]. Hence, sex differences in RMR might not have been detectable under the conditions of this study.

### Previous measurements and allometry

In the current study, we compared badger RMR values with a range of predictions. Metabolic rate of the European badger has only previously been measured in a single animal (during summer months, mean body mass: 10.30 kg) which recorded a lower value than that predicted for mammals with a BMR of 1389 kJ⋅d^−1^ (17.5% lower than the Kleiber (1961) [40] prediction (Eq 4)) [25, 26]. This is equivalent to a food intake of approximately 110 earthworms (*Lumbricus terrestris*) per day (with an energetic value of 12.59 kJ per average 4.27 g worm) [66]. In the current study, we found that the majority of individual badger RMR readings were higher than that reported by Iversen (1970, 1972) [25, 26]. Our values were also significantly greater than predicted for large mustelids (Eq 3) during summer, but not in autumn or winter. Morphologically, badgers are long and wedge-shaped, with a low-slung and elongated skeleton that conforms to general mustelid proportions [63]. Although less pronounced than in smaller mustelid species, it is possible that this leads to an energetically disadvantageous surface-to-volume ratio and may contribute to the relatively high RMR values in the present study.

### Future work

It is important that experimental procedures do not bias results by influencing the metabolic rate of animals as they are being measured. Lower than expected BMR (compared with [25]) has been reported in the black-footed ferret (*M. nigripes*) and Siberian polecat (*M. eversmannii*), which was attributed to handling procedures that reduced stress. In the present study, minimization of stress was an important consideration and consequently captured badgers remained inactive for long periods, although a passive or reactive [67] stress response cannot be discounted. Trapping has previously been shown to influence levels of fecal cortisol metabolites in badgers [68].

Variation in measurements can also arise if the animals were not in a truly post-absorptive state. We were not able to determine the time badgers had spent in traps before collection from the field, which could have ranged between two and 18 hours before RMR was measured. Hence, although some food could have remained in the digestive tract these potential sources of error are likely to have been random with respect to age, sex and season and so should not have influenced our results. The current study measured RMR whilst each of the previously published allometric predictions are of BMR and thus not directly comparable. Nevertheless, our measured values were closest to those predicted for large mustelids, and did not differ significantly during autumn and winter.

Finally, the full shape of the $\dot{\text{V}}{\text{O}}_{2}$-temperature response curve should be clarified. In Iversen (1970) [26], the measurements used to calculate BMR were recorded at 22°C. When the temperature decreased to 18–16°C there was an increase in metabolic rate indicating that this range was below the lower critical point. In the present study, we selected 20 ± 1°C based on our measurements of a captive badger at a range of ambient temperatures. Clearly there is a need for data from more individuals at different times of year to account for possible age and season related influences on the TNZ [69, 70], such as those resulting from changes in body composition and levels of subcutaneous fat.

## Conclusions

In cold regions, badgers are known to become less active during winter when they spend longer periods within their setts, often huddling [71], in what is a temperature buffered microclimate [72]. This reduced activity, termed winter lethargy, is associated with several physiological changes including depressed thyroxine plasma concentrations, increased utilisation of adipose reserves, and reductions in body temperature [19, 52, 73]. Possible environmental cues for these behavioural and physiological adaptations include photoperiod, winter temperature, and precipitation [18, 19, 74, 75]. In the present study, we provide evidence that a reduction in RMR occurs in winter, suggesting that badgers adopt an energy conservation strategy at times when thermoregulatory challenges are greatest and food is least available. Such adaptations may also be of importance when making allometric predictions of metabolic rate.

## Supporting Information

S1 Dataset Compressed.zip containing data used in anaesthesia, body mass, RMR, and allometric prediction analyses. (ZIP)
